# Postbiotics lower adipose tissue MHCII inflammation and blood glucose in obese mice, dependent on sex

**DOI:** 10.14814/phy2.70925

**Published:** 2026-05-21

**Authors:** Angela M. Schmidt, Amee M. Scribe, Nicole G. Barra, Brittany M. Duggan, Han Fang, Dana Kukje Zada, Jonathan D. Schertzer

**Affiliations:** ^1^ Department of Biochemistry and Biomedical Sciences McMaster University Hamilton Ontario Canada; ^2^ Farncombe Family Digestive Health Research Institute McMaster University Hamilton Ontario Canada; ^3^ Centre for Metabolism, Obesity and Diabetes Research McMaster University Hamilton Ontario Canada

**Keywords:** biological sex, blood glucose, inflammation, obesity, postbiotics

## Abstract

Obesity is characterized by compartmentalized inflammation in metabolic tissues and risk of dysglycemia, which can be influenced by bacterial components and metabolites (i.e., postbiotics). The postbiotic muramyl dipeptide (MDP) improves blood glucose control in obese male, but not female, mice. The immune response linking MDP to blood glucose was not known. We hypothesized that MDP lowers class II major histocompatibility complex (MHCII) immunity in a sex‐ and tissue‐dependent manner, and that lower MHCII immunity in adipose tissue correlates with improved blood glucose control in obese male mice. We showed that diet‐induced obesity increased the transcript levels of multiple components of MHCII immunity in adipose tissue of obese male and female mice, which positively correlated with increased fasting blood glucose. We found that 5 weeks of MDP injections lowered transcript levels of MHCII surface expression and T‐cell activation markers in adipose tissue of obese male mice, with minimal changes in the liver or in obese female mice. Lower MHCII inflammatory markers in adipose tissue positively correlated with improved glucose tolerance, but not fasting blood glucose, in obese male mice. Therefore, postbiotic‐induced sex‐specific improvements blood glucose control are linked to lower transcription of MHCII inflammation in adipose tissue of obese male mice.

## Graphical abstract

Postbiotics lower adipose tissue MHCII inflammation and improve blood glucose control in male mice during obesity.

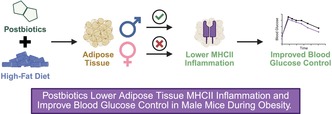

## 1 INTRODUCTION

Obesity is a chronic disease characterized by the abnormal or excessive accumulation of body fat (i.e., adiposity), which increases the risk of developing insulin resistance and impaired blood glucose control (Kahn et al., 2006; Wharton et al., 2020). Impaired blood glucose control can present as higher fasting blood glucose or higher blood glucose after an acute glucose load (i.e., glucose tolerance test) (Punthakee et al., 2018). The presentation of impaired blood glucose control during obesity varies by biological sex; females are more likely to develop impaired glucose tolerance (IGT) and less likely to develop impaired fasting glucose (IFG) than males (Link & Reue, 2017; Unwin et al., 2002). Both IGT and IFG increase the risk of developing type 2 diabetes (T2D) and cardiovascular disease (CVD), with some studies suggesting that IGT increases the risk of developing hypertension, coronary heart disease, and stroke more than IFG (Cai et al., 2020; Unwin et al., 2002). Improving IGT and IFG in individuals living with obesity can decrease the risk of developing comorbid chronic diseases and reduce all‐cause mortality (Diallo et al., 2025). New therapeutic agents that consider sex differences in blood glucose control are needed to improve IGT and IFG in males and females during obesity (Tannenbaum et al., 2017).

Obesity is associated with chronic, compartmentalized inflammation in tissues that regulate blood glucose, like the liver and adipose tissue, which is largely characterized by increased pro‐inflammatory macrophage accumulation and cytokine secretion (Khanna et al., 2022; Sun & Karin, 2012). Pro‐inflammatory cytokines, along with other secreted factors, can directly impair insulin signaling in these tissues and, depending on the primary site of insulin resistance, may promote the development of IFG or IGT (Abdul‐Ghani et al., 2006; Rohm et al., 2022). Biological sex influences immunity, with female adipose tissue demonstrating reduced pro‐inflammatory macrophage accumulation compared to male adipose tissue during obesity (Braga Tibaes et al., 2024). Lowering liver and adipose tissue inflammation can improve blood glucose control during obesity (Rohm et al., 2022). However, changes in general markers of inflammation, like cytokine and chemokine levels, do not always correlate with changes in IGT and IFG during obesity, indicating that other more specific immune responses may be involved (Duggan et al., 2022; Weiss et al., 2017).

Recently, the class II major histocompatibility complex (MHCII) immune response has been shown to play a critical role in the progression of adipose tissue inflammation during obesity, promoting pro‐inflammatory macrophage accumulation and cluster of differentiation 4 positive (CD4+) T‐cell activation, which contribute to adipose and hepatic insulin resistance (Cho et al., 2014, 2016; Deng et al., 2013, 2017; Xiao et al., 2016; Zhang et al., 2016). The link between the MHCII immune response in the liver and dysglycemia during obesity is less established, with some studies suggesting that activated Kupffer cells, which express MHCII on their surface, are key contributors to hepatic insulin resistance (Lanthier et al., 2010). Tissue‐resident immune cells and parenchymal cells in metabolic tissues, which can engage in MHCII‐mediated CD4+ T‐cell activation, directly respond to sex hormones, namely estradiol, progesterone, and androgens (Sharma et al., 2025). Sex hormone receptor signaling contributes to dynamic sex differences in immune responses like macrophage activation, pro‐inflammatory cytokine secretion, and surface receptor expression, across the life course (Hoffmann et al., 2023; Klein & Flanagan, 2016). Therefore, it is important to understand how obesity influences the MHCII immune response in metabolic tissues, and how this is linked to sex differences in IGT and IFG. It is plausible that compartmentalized MHCII inflammation in metabolic tissues may be contributing to sex differences in impaired blood glucose control during obesity.

Obesity is associated with changes in intestinal bacteria (i.e., microbiota) composition, which can contribute to the development of inflammation, insulin resistance, and dysglycemia (Anhê et al., 2023; Caricilli & Saad, 2013; Foley et al., 2018). Certain microbiota‐derived components or metabolites, termed postbiotics, can improve insulin resistance and blood glucose control (Anhê et al., 2021; Cavallari et al., 2017; Duggan et al., 2022; Pomié et al., 2016). We have shown that the postbiotic muramyl dipeptide (MDP), a bacterial cell wall component, improves blood glucose control in male, but not female, mice during obesity (Duggan et al., 2022). However, these sex‐specific improvements in glucose tolerance did not correlate with the widespread lowering of adipose tissue inflammation, which occurred in obese male and female mice (Duggan et al., 2022). Here, we aimed to determine the specific immune response that underpins the ability of MDP to improve glucose tolerance in a sex‐specific manner during obesity. MDP activates the nucleotide‐binding oligomerization domain‐containing protein 2 (NOD2) receptor, which is expressed by cells in both the liver and adipose tissue (Cavallari et al., 2017). MDP‐mediated NOD2 activation can increase MHCII inflammation in macrophages and dendritic cells, but its effect on MHCII inflammation in tissues that regulate blood glucose has yet to be determined (Cooney et al., 2010; Guryanova & Khaitov, 2021; Mansilla et al., 2024).

Here, we show that MDP lowers adipose tissue MHCII inflammation and NOD2 receptor expression in a sex‐dependent manner. Lower MHCII inflammation and NOD2 receptor expression in adipose tissue correlated with improved glucose tolerance in obese male, but not female, mice. These findings support the concept that postbiotic‐mediated improvements in blood glucose tolerance are linked to lower adipose tissue MHCII inflammation in a tissue‐ and sex‐dependent mechanism.

## 2 MATERIALS AND METHODS

### 2.1 Mice and experimental design

**FIGURE 1:**
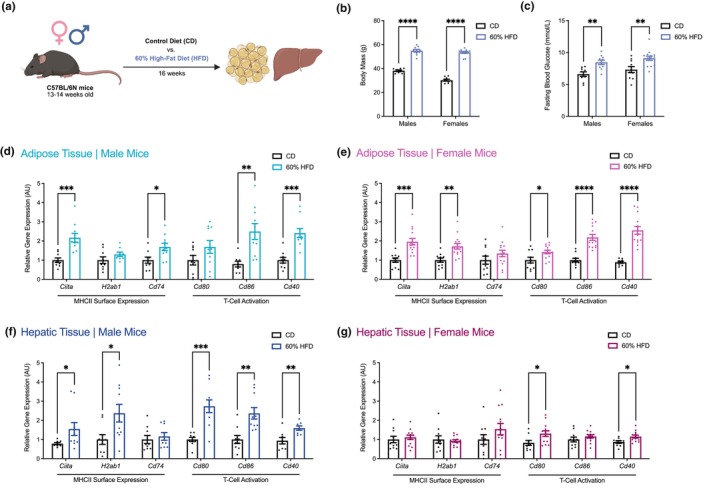
Obesity increases markers of MHCII inflammation in a tissue‐ and sex‐dependent manner. (a) Male and female C57BL/6N mice were fed a control diet (CD) or 60% high‐fat diet (HFD) for 16 weeks, after which adipose and hepatic tissues were collected and stored at −80°C. (b) Body mass comparison at 16 weeks for male and female mice fed a 60% HFD compared to CD. Body mass is reported in grams (g). (c) Blood glucose level comparison at 14–16 weeks for the same male and female mice after 12 or 4 h of fasting, respectively. Fasting blood glucose is reported in millimoles per liter (mmol/L). Quantification of the relative changes in mRNA levels of markers of class II major histocompatibility complex (MHCII) inflammation (*Ciita*, *H2ab1*, *Cd74*, *Cd80*, *Cd86*, and *Cd40*) in adipose and hepatic tissues collected from (d, f) male and (e, g) female mice fed a 60% HFD compared to CD. Data is expressed as mean ± SEM (*n* = 8–14 per group). Each dot is a separate mouse. Statistical significance was measured as *p* < 0.05 after testing for normality and equal variance and then applying an unpaired *t*‐test, an unpaired *t*‐test with Welch's correction, or a Mann–Whitney test as appropriate. Statistical significance is indicated with asterisks (*p < 0.05; **p < 0.01; ***p < 0.001; ****p < 0.0001).

All mouse procedures were approved by the McMaster University Animal Research Ethics Board (AREB) in compliance with the Canadian Council of Animal Care's (CCAC) recommendations. Mice were housed at room temperature (23°C) in groups of up to 5 mice per cage and kept under 12‐h light–dark conditions with ad libitum access to water and food. Thirteen‐ to fourteen‐week‐old male and female C57BL/6N mice (Taconic, #C57BL/6NTac) were either placed on a control diet (Envigo, #8640 Teklad 22/5) or given a 60% high‐fat diet (Research Diets, #D12429) for 16 weeks (Figure 1a). During weeks 14–16, fasting blood glucose was measured. At the scientific endpoint, body mass was measured, and liver and gonadal white adipose tissue (gWAT) were collected, flash frozen in liquid nitrogen, and stored at −80°C until further analyses.

In a previous publication, 9‐ to 12‐week‐old male and female wild‐type mice (The Jackson Laboratory, #009380) were switched from a control diet (Envigo, #8640 Teklad 22/5) to a 60% high‐fat diet (Research Diets, #D12429) and injected with MDP (100 μg, i.p.; InvivoGen, #tlrl‐mdp) or saline 4 days per week for 5 weeks (Figure 3a). At the end of the 4th week, a glucose tolerance test (GTT) was performed as previously described (Anhê et al., 2022; Duggan et al., 2022). At the scientific endpoint, body mass and fasting blood glucose were measured, and liver and gWAT were collected, flash frozen in liquid nitrogen, and stored at −80°C until further analyses. In Figures 3, 4, 5, we conducted additional analyses of transcript markers of MHCII immunity and NOD2 receptor activation in existing tissues from wild‐type mice from this previous publication (Duggan et al., 2022). We also reanalyzed blood glucose data from the same wild‐type mice (Duggan et al., 2022).

### 2.2 Liver and adipose tissue gene expression

RNA was isolated from hepatic and adipose tissues via mechanical homogenization at 4.5 m/s for 60 s using a FastPrep‐24 tissue homogenizer (MP Biomedicals) and glass beads, followed by phenol‐chloroform extraction. RNA concentrations were calculated using the NanoDrop One™ Microvolume UV–Vis Spectrophotometer (Thermo Fisher Scientific). RNA samples (400–800 ng adipose tissue; 2000 ng hepatic tissue) were treated with DNase I (Invitrogen, #18068015) and converted to cDNA using the SuperScript IV reverse transcriptase kit (Invitrogen, #18090200). qPCR was performed using TaqMan Assays (Thermo Fisher Scientific) with AmpliTaq Gold DNA polymerase (Applied Biosystems, #4311806) on a Rotor‐Gene Q instrument (Qiagen). Amplification consisted of 50 cycles at 95°C for 10 s and 58°C for 45 s. Levels of target gene expression were calculated using the 2^−ΔΔCT^ method (Livak & Schmittgen, 2001) and normalized to ribosomal protein lateral stalk subunit P0 (*Rplp0*) expression levels. Target gene expression levels were divided by the mean of the control group, setting controls to 1 for statistical analysis.

Transcript markers of MHCII immunity included: class II major histocompatibility complex transactivator (*Ciita*; Mm00482914_m1), H2A.B variant histone 1 (*H2ab1*; Mm00439216_m1), CD74 molecule (*Cd74*; Mm00658576_m1), CD80 molecule (*Cd80*; Mm00711660_m1), CD86 molecule (*Cd86*; Mm00444540_m1), and CD40 molecule (*Cd40*; Mm00441891_m1). *Ciita* is considered the master transcriptional regulator of MHCII inflammation, exhibiting a high specificity for genes involved in MHCII‐mediated antigen presentation (LeibundGut‐Landmann et al., 2004; Nakamura, 2014). *H2ab1* is located within the major functional MHCII gene region in mice (H2‐A region on chromosome 17), encoding for the β‐chain of the I‐A molecule, which enables antigen presentation to CD4+ T cells (Ubogu et al., 2024). *Cd74* encodes for the invariant chain, which assists in MHCII complex assembly and trafficking to the cell membrane (Schröder, 2016). *Cd80*, *Cd86*, and *Cd40* encode for co‐stimulatory molecules involved in MHCII‐mediated CD4+ T‐cell activation (Elgueta et al., 2009; Sharpe, 2009). Nucleotide binding oligomerization domain containing 2 (*Nod2*; Mm00467543_m1) encodes for NOD2, an innate immune receptor that detects bacterial cell wall components like MDP (Cavallari et al., 2017). Transcript markers of general inflammation included: c‐c motif chemokine ligand 2 (*Ccl2*; Mm00441242_m1), tumor necrosis factor (*Tnf*; Mm00443258_m1), interleukin 6 (*Il6*; Mm00446190_m1), and interleukin 1 beta (*Il1b*; Mm00434228_m1). These cytokines and chemokines play a key role in the development and progression of chronic adipose tissue inflammation during obesity (Uti et al., 2025). Transcript markers of immune cells included: adhesion G protein‐coupled receptor E1 (*Adgre1*; Mm00802529_m1), integrin subunit alpha X (*Itgax*; Mm00498698_m1), CD4 molecule (*Cd4*; Mm00442754_m1), and CD8 subunit alpha (*Cd8a*; Mm01182107_g1). These cell surface markers are commonly used for the identification of macrophages, dendritic cells, and T‐cell subsets in mice, respectively (Haugstøyl et al., 2023; Sundara Rajan & Longhi, 2016).

### 2.3 Statistical analyses

All statistical analyses were performed using the GraphPad Prism Software (Version 11.0.1). Data was first assessed for outliers using the Grubb's test and the robust regression and outlier removal (ROUT) test; outliers identified by either test were excluded. Data was then assessed for normality using the D'Agostino and Pearson, Anderson‐Darling, Shapiro–Wilk, and Kolmogorov–Smirnov tests. For body mass, fasting blood glucose, and qPCR analyses, if data passed all normality tests, an unpaired *t*‐test was conducted. If variances were not equal, Welch's correction was included. If data failed any normality test, a Mann–Whitney test was conducted. Data was expressed as mean ± SEM and statistical significance was measured as *p* < 0.05. For correlation analyses, if data passed all normality tests, a Pearson correlation test was conducted, generating a Pearson correlation coefficient (*r*). If data failed any normality test, a Spearman rank correlation test was conducted, generating a Spearman rank correlation coefficient (ρ). Statistical significance was defined as *p* < 0.05.

## 3 RESULTS

### 3.1 Obesity increases MHCII inflammation in a tissue‐ and sex‐dependent manner

Male and female mice fed a 60% high‐fat diet (HFD) for 16 weeks had a significant increase in body mass and fasting blood glucose compared to mice fed a control diet (CD) (Figure 1a–c). Obesity significantly increased the mRNA levels of MHCII surface expression and T‐cell activation markers in the adipose tissue of male (*Ciita*, *Cd74*, *Cd86*, and *Cd40*) and female (*Ciita*, *H2ab1*, *Cd80*, *Cd86*, and *Cd40*) mice (Figure 1d,e). In hepatic tissue, the effects of obesity on MHCII inflammation were sex dependent. In male mice, obesity increased the mRNA levels of MHCII surface expression (*Ciita* and *H2ab1*) and T‐cell activation (*Cd80*, *Cd86*, and *Cd40*) markers (Figure 1f). However, in female mice, obesity only increased the mRNA levels of T‐cell activation markers (*Cd80* and *Cd40*) (Figure 1g). This suggests that increases in MHCII inflammation during diet‐induced obesity in mice are both tissue‐ and sex‐dependent.

### 3.2 Higher MHCII inflammation in adipose tissue, but not in hepatic tissue, correlates with higher fasting blood glucose

**FIGURE 2:**
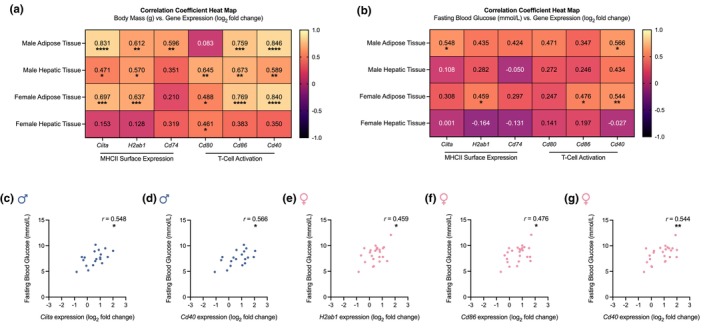
Obesity increases MHCII inflammation in adipose tissue, which correlates with higher fasting blood glucose. (a) Correlations between body mass and mRNA levels of markers of class II major histocompatibility complex (MHCII) inflammation (*Ciita*, *H2ab1*, *Cd74*, *Cd80*, *Cd86*, and *Cd40*) in adipose and hepatic tissues collected from male and female mice fed either a 60% high‐fat diet (HFD) or control diet (CD) for 16 weeks. (b) Correlations between fasting blood glucose and mRNA levels of markers of MHCII inflammation (*Ciita*, *H2ab1*, *Cd74*, *Cd80*, *Cd86*, and *Cd40*) in adipose and hepatic tissues collected from the same male and female mice. Statistically significant correlations between fasting blood glucose and gene expression of (c) *Ciita* (*r* = 0.548; 95% CI [0.125, 0.803]), (d) *Cd40* (*r* = 0.566; 95% CI [0.151, 0.812]), (e) *H2ab1* (*r* = 0.459; 95% CI [0.068, 0.727]), (f) *Cd86* (*r* = 0.476; 95% CI [0.090, 0.738]), and (g) *Cd40* (*r* = 0.544; 95% CI [0.180, 0.777]) in (c, d) male and (e–g) female adipose tissue. Body mass is reported in grams (g). Fasting blood glucose is reported in millimoles per liter (mmol/L). Gene expression is reported as log_2_ fold change. Correlation coefficients were calculated in all samples (*n* = 17–24 per group) after testing for normality and then applying Pearson's or Spearman's rank correlation as appropriate. Each dot is a separate mouse. Statistical significance was defined as *p* < 0.05 and is indicated with asterisks (*p < 0.05; **p < 0.01; ***p < 0.001; ****p < 0.0001).

We assessed if obesity's tissue‐ and sex‐dependent effects on MHCII inflammation are correlated with its effects on body mass and fasting blood glucose. There were significant positive correlations between body mass and mRNA levels of MHCII surface expression and T‐cell activation markers in male adipose tissue (*Ciita*, *H2ab1*, *Cd74*, *Cd86*, and *Cd40*), female adipose tissue (*Ciita*, *H2ab1*, *Cd80*, *Cd86*, and *Cd40*), and male hepatic tissue (*Ciita*, *H2ab1*, *Cd80*, *Cd86*, and *Cd40*) (Figure 2a). In female hepatic tissue, only *Cd80* expression showed a significant positive correlation with body mass (Figure 2a). There were no significant correlations between fasting blood glucose and the mRNA levels of any MHCII inflammatory marker in hepatic tissue (Figure 2b). However, the expression of *Ciita* (Figure 2c) and *Cd40* (Figure 2d) in male adipose tissue, and *H2ab1* (Figure 2e), *Cd86* (Figure 2f), and *Cd40* (Figure 2g) in female adipose tissue, showed a significant positive correlation with fasting blood glucose. These data suggest that increases in adipose tissue, but not liver, MHCII inflammation during diet‐induced obesity in mice may be linked to sex differences in blood glucose control.

### 3.3 MDP lowers MHCII inflammation in the adipose tissue of male mice during obesity

**FIGURE 3:**
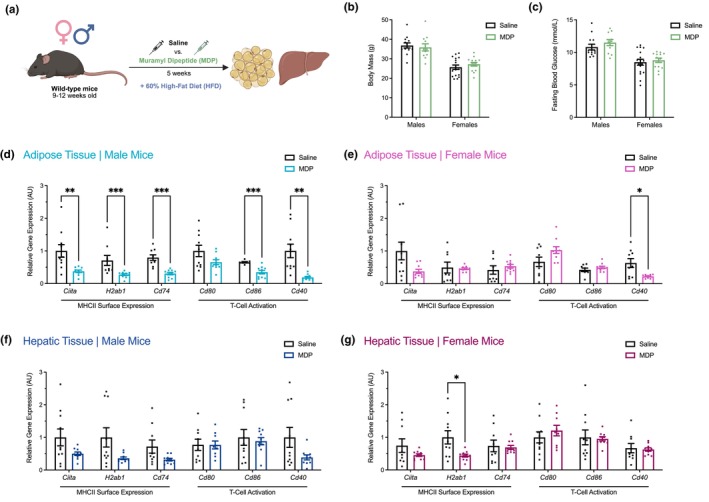
The postbiotic muramyl dipeptide lowers markers of MHCII inflammation in a tissue‐ and sex‐dependent manner during obesity. (a) Male and female wild‐type mice were fed a 60% high‐fat diet (HFD) and injected with either muramyl dipeptide (MDP) or saline 4 days per week for 5 weeks, after which adipose and hepatic tissues were collected and stored at −80°C. (b) Body mass comparison at 5 weeks for obese male and female mice injected with MDP compared to saline. Body mass is reported in grams (g). (c) Blood glucose level comparison at 5 weeks for the same male and female mice after 6 h of fasting. Fasting blood glucose is reported in millimoles per liter (mmol/L). Quantification of the relative changes in mRNA levels of markers of class II major histocompatibility complex (MHCII) inflammation (*Ciita*, *H2ab1*, *Cd74*, *Cd80*, *Cd86*, and *Cd40*) in adipose and hepatic tissues collected from obese (d, f) male and (e, g) female mice injected with MDP compared to saline. Data is expressed as mean ± SEM (*n* = 7–17 per group). Each dot is a separate mouse. Statistical significance was measured as *p* < 0.05 after testing for normality and equal variance and then applying an unpaired *t*‐test, an unpaired *t*‐test with Welch's correction, or a Mann–Whitney test as appropriate. Statistical significance is indicated with asterisks (*p < 0.05; **p < 0.01; ***p < 0.001; ****p < 0.0001).

Male and female wild‐type mice fed a 60% HFD and injected with MDP for 5 weeks had no significant difference in body mass or fasting blood glucose compared to 60% HFD‐fed saline‐injected mice (Figure 3a–c). We have previously shown that 5 weeks of MDP injections had sex‐dependent effects on blood glucose tolerance, where MDP lowered blood glucose during a GTT in obese male, but not female, mice (Duggan et al., 2022). MDP significantly lowered the mRNA levels of MHCII inflammatory markers in the adipose tissue of male mice (*Ciita*, *H2ab1*, *Cd74*, *Cd86*, and *Cd40*) (Figure 3d). However, MDP had a limited effect on MHCII inflammation in the adipose tissue of female mice, where only *Cd40* mRNA levels were lower (Figure 3e). Moreover, the effect of MDP on MHCII inflammation in hepatic tissue was minimal, as there were no changes in male mice and only *H2ab1* mRNA levels were lower in female mice (Figure 3f,g). This suggests that the effect of MDP on MHCII inflammation during obesity is largely compartmentalized in adipose tissue and dependent on biological sex.

### 3.4 MDP lowers NOD2 receptor expression in the adipose tissue of male mice during obesity

**FIGURE 4:**
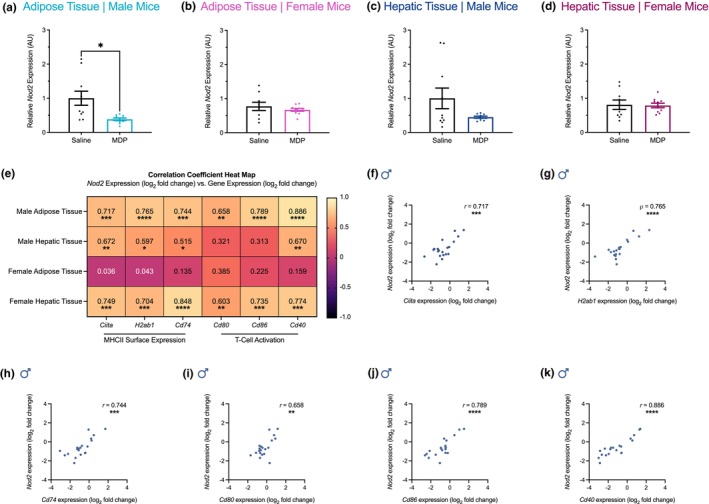
The postbiotic muramyl dipeptide lowers NOD2 receptor expression in male adipose tissue, which correlates with lower MHCII inflammation during obesity. Quantification of the relative changes in mRNA levels of *Nod2* in adipose and hepatic tissues collected from obese (a, c) male and (b, d) female mice injected with muramyl dipeptide (MDP) compared to saline. Data is expressed as mean ± SEM (*n* = 9–10 per group). Statistical significance was measured as *p* < 0.05 after testing for normality and equal variance and then applying an unpaired *t*‐test, an unpaired *t*‐test with Welch's correction, or a Mann–Whitney test as appropriate. (e) Correlations between mRNA levels of *Nod2* and class II major histocompatibility complex (MHCII) inflammatory markers (*Ciita*, *H2ab1*, *Cd74*, *Cd80*, *Cd86*, and *Cd40*) in adipose and hepatic tissues collected from the same male and female mice. Statistically significant correlations between *Nod2* expression and gene expression of (f) *Ciita* (*r* = 0.717; 95% CI [0.401, 0.880]), (g) *H2ab1* (ρ = 0.765; 95% CI [0.476, 0.905]), (h) *Cd74* (*r* = 0.744; 95% CI [0.450, 0.893]), (i) *Cd80* (*r* = 0.658; 95% CI [0.303, 0.852]), (j) *Cd86* (*r* = 0.789; 95% CI [0.533, 0.913]), (k) *Cd40* (*r* = 0.886; 95% CI [0.729, 0.954]) in male adipose tissue. Gene expression is reported as log_2_ fold change. Correlation coefficients were calculated in all samples (*n* = 16–20 per group) after testing for normality and then applying Pearson's or Spearman's rank correlation as appropriate. Each dot is a separate mouse. Statistical significance was defined as *p* < 0.05 and is indicated with asterisks (*p < 0.05; **p < 0.01; ***p < 0.001; ****p < 0.0001).

MDP lowered the mRNA levels of *Nod2* in the adipose tissue of male mice (Figure 4a). MDP did not significantly change the mRNA levels of *Nod2* in the adipose tissue of female mice, or in the hepatic tissue of male or female mice (Figure 4b–d). This suggests that the effect of MDP on NOD2 receptor expression, like its effect on MHCII inflammation, is compartmentalized in adipose tissue and dependent on biological sex. We next assessed whether MDP's sex‐dependent effects on NOD2 receptor expression correlated with its sex‐dependent effects on MHCII inflammation in adipose tissue or hepatic tissue (Figure 4e). The expression of *Ciita* (Figure 4f), *H2ab1* (Figure 4g), *Cd74* (Figure 4h), *Cd80* (Figure 4i), *Cd86* (Figure 4j), and *Cd40* (Figure 4k) in male adipose tissue showed a significant positive correlation with *Nod2* expression. Moreover, there were no significant correlations between *Nod2* expression and mRNA levels of MHCII inflammatory markers in female adipose tissue (Figure 4e). Interestingly, there were significant positive correlations between *Nod2* expression and mRNA levels of MHCII inflammatory markers in male hepatic tissue (*Ciita*, *H2ab1*, *Cd74*, and *Cd40*) and female hepatic tissue (*Ciita*, *H2ab1*, *Cd74*, *Cd80*, *Cd86*, and *Cd40*) (Figure 4e). Together, these data suggest that MDP‐mediated lowering of MHCII inflammation may be linked to MDP‐mediated lowering of NOD2 receptor expression in male adipose tissue during obesity.

### 3.5 MDP‐mediated lowering of MHCII inflammation and NOD2 receptor expression in male adipose tissue correlates with improvements in blood glucose tolerance in obese mice

**FIGURE 5:**
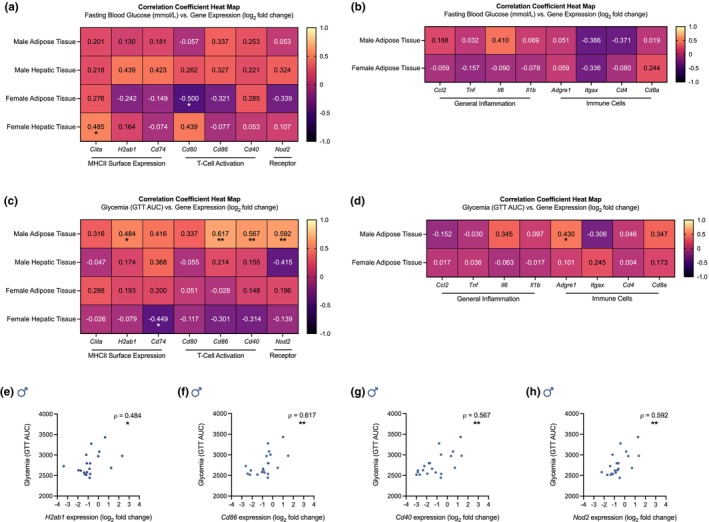
Postbiotic lowering of MHCII inflammation and NOD2 receptor expression in male adipose tissue correlates with improvements in glucose tolerance during obesity. Correlations between fasting blood glucose and mRNA levels of (a) class II major histocompatibility complex (MHCII) inflammatory markers (*Ciita*, *H2ab1*, *Cd74*, *Cd80*, *Cd86*, and *Cd40*), (a) *Nod2*, (b) general inflammatory markers (*Ccl2*, *Tnf*, *Il6*, and *Il1b*), and (b) immune cell markers (*Adgre1*, *Itgax*, *Cd4*, and *Cd8a*) in adipose and hepatic tissues collected from obese male and female mice injected with either muramyl dipeptide (MDP) or saline for 5 weeks. Correlations between glycemia and mRNA levels of (c) MHCII inflammatory markers (*Ciita*, *H2ab1*, *Cd74*, *Cd80*, *Cd86*, and *Cd40*), (c) *Nod2*, (d) general inflammatory markers (*Ccl2*, *Tnf*, *Il6*, and *Il1b*), and (d) immune cell markers (*Adgre1*, *Itgax*, *Cd4*, and *Cd8a*) in adipose and hepatic tissues collected from the same male and female mice. Statistically significant correlations between glycemia and gene expression of (e) *H2ab1* (ρ = 0.484; 95% CI [0.038, 0.769]), (f) *Cd86* (ρ = 0.617; 95% CI [0.226, 0.836), (g) *Cd40* (ρ = 0.567; 95% CI [0.152, 0.812]), and (h) *Nod2* (ρ = 0.592; 95% CI [0.189, 0.824]) in male adipose tissue. Fasting blood glucose is reported in millimoles per liter (mmol/L). Glycemia is reported as area under the curve (AUC) during a glucose tolerance test (GTT). Gene expression is reported as log_2_ fold change. Correlation coefficients were calculated in all samples (*n* = 16–31 per group) after testing for normality and then applying Pearson's or Spearman's rank correlation as appropriate. Each dot is a separate mouse. Statistical significance was defined as *p* < 0.05 and is indicated with asterisks (*p < 0.05; **p < 0.01; ***p < 0.001; ****p < 0.0001).

We assessed if the sex‐dependent effects of MDP on MHCII inflammation and NOD2 receptor expression in adipose tissue or hepatic tissue were correlated with its effects on fasting blood glucose (Figure 5a). Beyond a negative correlation between adipose tissue mRNA levels of *Cd80* and fasting blood glucose in female mice, there were no significant correlations between fasting blood glucose and the mRNA levels of *Nod2*, immune cell markers, general inflammatory markers, or MHCII inflammatory markers in male or female adipose tissue (Figure 5a,b). Moreover, significant positive correlations between fasting blood glucose and the mRNA levels of *Nod2* and MHCII inflammatory markers in hepatic tissue were limited to *Ciita* in female hepatic tissue (Figure 5a). These results suggest that compartmentalized changes in adipose tissue NOD2 receptor expression and MHCII inflammation following MDP treatment are not linked to changes in fasting blood glucose. Therefore, we assessed whether they were linked to changes in blood glucose tolerance, which were sex dependent.

**FIGURE 6:**
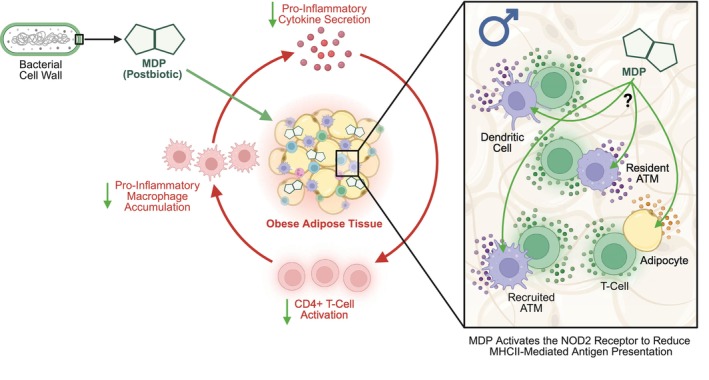
The postbiotic muramyl dipeptide may activate the NOD2 receptor to lower MHCII‐mediated CD4+ T‐cell activation in adipose tissue and improve blood glucose control in a sex‐dependent manner during obesity. The postbiotic muramyl dipeptide (MDP) lowers nucleotide‐binding oligomerization domain‐containing protein 2 (NOD2) receptor expression in adipose tissue from male, but not female, mice during obesity. Lower NOD2 receptor expression positively correlates with both lower class II major histocompatibility complex (MHCII) inflammation and improved blood glucose tolerance in male mice. MHCII immunity plays a critical role in the development and progression of adipose tissue inflammation during obesity, promoting cluster of differentiation 4 positive (CD4+) T‐cell activation and pro‐inflammatory macrophage accumulation, which contribute to adipose and hepatic insulin resistance. Therefore, MDP may be activating the NOD2 receptor to reduce MHCII‐mediated CD4+ T‐cell activation and pro‐inflammatory macrophage accumulation in adipose tissue and improve blood glucose control in a sex‐dependent manner during obesity. ATM is an abbreviation for adipose tissue macrophage.

We have previously shown that MDP treated male, but not female, wild‐type mice fed a 60% HFD had improved glucose tolerance, with a significantly lower area under the curve (AUC) for blood glucose over time during a GTT (Duggan et al., 2022). We have also shown that the mRNA levels of general inflammatory markers and the T‐cell markers *Cd4* and *Cd8a* were lower in the adipose tissue of both male and female wild‐type mice after a 60% HFD and MDP injections (Duggan et al., 2022). MDP‐treated male and female mice fed a 60% HFD showed no significant change in the mRNA levels of *Itgax* (data not shown). However, the mRNA levels of *Adgre1* were lower in the adipose tissue of MDP‐treated male, but not female, 60% HFD‐fed mice (Duggan et al., 2022). Here, we assessed if MDP's sex‐dependent effects on AUC during a GTT are correlated with its sex‐dependent effects on NOD2 receptor expression and MHCII inflammation in adipose tissue or hepatic tissue (Figure 5c). We also assessed if MDPs effects on general inflammatory or immune cell markers in adipose tissue correlated with its sex‐dependent effects on AUC during a GTT (Figure 5d). The expression of *H2ab1* (Figure 5e), *Cd86* (Figure 5f), *Cd40* (Figure 5g), and *Nod2* (Figure 5h) in male adipose tissue showed a significant positive correlation with AUC during a GTT. There were no significant correlations between AUC during a GTT and mRNA levels of *Nod2* or MHCII inflammatory markers in female adipose tissue (Figure 5c). Moreover, significant correlations between AUC during a GTT and mRNA levels of general inflammatory or immune cell markers in adipose tissue were limited to *Adgre1* in male adipose tissue (Figure 5d). In hepatic tissue, there were no significant positive correlations between the mRNA levels of *Nod2* or any MHCII inflammatory marker and glycemia (Figure 5c). These results suggest that the sex‐dependent effects of MDP on blood glucose tolerance during obesity may be mediated, in part, by compartmentalized changes in adipose tissue MHCII inflammation, not general inflammation, in a NOD2 receptor‐mediated mechanism (Figure 6).

## 4 DISCUSSION

Obesity is associated with compartmentalized inflammation in the liver and adipose tissue that participates in interorgan communication that can impair blood glucose control (Khanna et al., 2022; Rohm et al., 2022; Sun & Karin, 2012). There are many potential triggers of inflammation during diet‐induced obesity, including dietary components like saturated fatty acids and glucose, or changes in the gut microbiota (Tanti et al., 2013). Typically, these potential triggers of inflammation engage innate immune pattern recognition receptors (PRRs) in metabolic tissues to promote pro‐inflammatory cytokine production, immune cell recruitment, and metabolic inflammation (Tanti et al., 2013). However, not all PRRs lead to increased metabolic inflammation. The PRRs nucleotide‐binding oligomerization domain‐containing protein 1 (NOD1) and NOD2 have contrasting effects on blood glucose control. NOD1 activation increases liver and adipose tissue inflammation and worsens blood glucose control, while NOD2 activation promotes immune tolerance in adipose tissue and improves blood glucose control (Rodrigues e‐Lacerda et al., 2023). MDP is a postbiotic that has been shown to engage the NOD2 receptor, interacting serine/threonine protein kinase 2 (RIPK2), and interferon regulatory factor 4 (IRF4) to reduce metabolic inflammation and improve glucose tolerance in obese mice (Cavallari et al., 2017, 2020; Duggan et al., 2022). The effects of MDP on glucose tolerance during obesity are dependent on biological sex and do not correlate with its effects on general markers of adipose tissue inflammation such as cytokine and chemokine levels (Duggan et al., 2022). Therefore, we aimed to determine the specific and compartmentalized immune response in metabolic tissues that correlates with MDP's sex‐dependent effects on glucose tolerance during obesity.

The MHCII immune response contributes to the progression of adipose tissue inflammation during obesity and is shown to drive adipose and hepatic insulin resistance (Cho et al., 2014, 2016; Deng et al., 2013; Xiao et al., 2016; Zhang et al., 2016). Obesity increases MHCII expression in many different adipose tissue cell types, including adipocytes, macrophages, and dendritic cells (Cho et al., 2014, 2016; Deng et al., 2013; Xiao et al., 2016). However, the effects of obesity on MHCII expression in the liver are less understood (Lanthier et al., 2010). Here, we showed that obesity increased transcript levels of MHCII inflammatory markers independent of biological sex in adipose tissue, but that in the liver, obesity preferentially increased specific MHCII inflammatory markers in male mice. Increases in MHCII inflammatory markers in adipose tissue, but not in the liver, significantly correlated with increases in fasting blood glucose. This suggests that, in females, obesity‐driven changes in MHCII expression may be compartmentalized in adipose tissue. Moreover, obesity‐driven increases in MHCII expression in the liver do not seem to be contributing to IFG, which males are more likely to develop than females (Link & Reue, 2017; Unwin et al., 2002). We next explored if and how the postbiotic MDP alters MHCII inflammation in specific metabolic tissues to exert its sex‐dependent effects on blood glucose tolerance during obesity. We found that the effects of MDP on MHCII inflammation were compartmentalized in adipose tissue during obesity, and that lower transcript levels of MHCII inflammatory markers were positively correlated with both lower transcript levels of *Nod2* and improved glucose tolerance in obese male mice. Together, these results suggest that sex differences in liver MHCII inflammation during obesity may not influence the effects of MDP on blood glucose control, but rather that MDP may engage the MHCII immune response by activating the NOD2 receptor in adipose tissue in a sex‐dependent manner to improve blood glucose tolerance (Figure 6).

Further research is needed to uncover the specific cell type(s) and/or secreted factors that lower MHCII inflammation with MDP treatment in obese adipose tissue and how this may improve blood glucose control. Both acute and chronic NOD2 activation by MDP can increase MHCII expression in macrophages and dendritic cells (Cooney et al., 2010; Guryanova & Khaitov, 2021; Mansilla et al., 2024). MHCII expression is increased in large adipocytes in obesity, which can stimulate CD4+ T cells in adipose tissue to secrete the pro‐inflammatory cytokine interferon gamma (IFNg), promoting macrophage recruitment and pro‐inflammatory M1 polarization (Deng et al., 2013; Xiao et al., 2016). Inhibiting adipocyte MHCII‐mediated antigen presentation has been shown to improve adipose tissue insulin resistance during obesity, in male mice and in mice where the sex was not specified (Deng et al., 2017; Zhang et al., 2016). Importantly, MDP administration in our model does not influence adiposity and adipocyte size (Duggan et al., 2022). Adipose tissue transcript levels of the macrophage marker *Adgre1* were significantly lower and positively correlated with improved glucose tolerance in obese male, but not female, mice treated with MDP. Therefore, lower adipose‐resident macrophage number may be an important contributing factor to MDP's sex‐dependent effects on adipose tissue MHCII inflammation and blood glucose control during obesity. However, MDP may also influence the local communication between immune cells that reside in adipose tissue and adipocytes to exert its beneficial effects on blood glucose control (Figure 6).

MDP only improved blood glucose tolerance in male mice, and further research is needed to uncover the factors that may be driving this sex‐dependent effect. During obesity, excess adipose tissue is deposited differently depending on biological sex; females tend to accumulate more subcutaneous fat and less visceral fat compared to males (Lemieux et al., 1993). Visceral adipose tissue contains more large adipocytes compared to subcutaneous adipose tissue and is therefore more insulin‐resistant (Ibrahim, 2010). Whether different adipose depots respond differentially to MDP has not been determined but is an important future direction. Considering that MHCII‐mediated antigen presentation during obesity is dependent on adipocyte size, it is possible that sex differences in body fat accumulation are contributing to the ability of MDP to reduce adipose tissue MHCII inflammation (Xiao et al., 2016). However, it is also possible that the effects of MDP on adipose tissue MHCII inflammation are being driven by inherent sex differences in innate and adaptive immunity (Manuel & Liang, 2021).

In the second study, MDP was administered by intraperitoneal injection rather than orally to bypass the gut barrier and any potential gut microbiota interactions and directly assess its systemic effects, as we have done previously (Cavallari et al., 2017, 2020; Rodrigues e‐Lacerda et al., 2026). Future work will focus on oral delivery systems of postbiotics that allow penetration of the gut barrier for specific postbiotics. The timing of HFD feeding is an important factor to consider in the inflammatory and non‐inflammatory routes to insulin resistance (Foley et al., 2018; Lee et al., 2011). The first study in this paper fed mice a HFD for 14–16 weeks, but the second study injected MDP in mice fed a HFD for 5 weeks. The difference in the duration of HFD exposure is a limitation that may influence the magnitude of metabolic dysfunction and the reliance on inflammation to promote impaired blood glucose control. However, 5 weeks of HFD feeding in mice is a sufficient duration of exposure that increases inflammation and exceeds the amount of time required for the gut microbiota to contribute to impaired blood glucose control (Foley et al., 2018). Another limitation is that we only measured transcript markers of inflammation in metabolic tissues.

In conclusion, we have shown that obesity increases MHCII inflammation in male and female adipose tissue and that postbiotic treatment with MDP lowers MHCII inflammation in male adipose tissue. Lower MHCII inflammation positively correlated with lower NOD2 receptor expression and improved blood glucose tolerance, but not fasting blood glucose, in obese male mice treated with MDP. These results highlight the importance of considering sex differences in modulating immune responses like MHCII when developing postbiotic treatments to improve blood glucose control during obesity.

## AUTHOR CONTRIBUTIONS

**Angela M. Schmidt:** Conceptualization; data curation; formal analysis. **Amee M. Scribe:** Data curation; formal analysis. **Nicole G. Barra:** Conceptualization; data curation; formal analysis; project administration. **Brittany M. Duggan:** Data curation. **Han Fang:** Data curation. **Dana Kukje Zada:** Data curation. **Jonathan D. Schertzer:** Conceptualization; data curation; funding acquisition; project administration; supervision.

## FUNDING INFORMATION

This work was supported by a Canadian Institutes of Health Research (CIHR) project grant (PJT‐191869). Angela M. Schmidt was supported by a CIHR CGS‐M student scholarship and MD/PhD award, as well as an Ontario Graduate Scholarship. Han Fang was supported by a Farncombe Family Digestive Health Research Fellowship and a Center for Metabolism, Obesity, and Diabetes Research fellowship. Dana Kukje Zada was supported by a Farncombe Family Digestive Health Research Studentship Award. Jonathan D. Schertzer holds a Canada Research Chair in Metabolic Inflammation.

## CONFLICT OF INTEREST STATEMENT

No conflicts of interest, financial or otherwise, are declared by the authors.

## ETHICS STATEMENT

All experiments in mice were approved by the McMaster University Animal Research Ethics Board (AREB) with approval number AUP‐23‐64, and experiments were conducted in accordance with the guidelines of the Canadian Council on Animal Care (CCAC).

## Data Availability

Data will be made available upon reasonable request.
